# Hemodialysis through persistent left superior vena cava

**DOI:** 10.4103/0972-5229.78223

**Published:** 2011

**Authors:** V. B. Kute, A. V. Vanikar, M. R. Gumber, P. R. Shah, K. R. Goplani, H. L. Trivedi

**Affiliations:** **From:** Department of Nephrology and Clinical Transplantation, Transfusion Services and Immunohematology, Institute of Kidney Diseases and Research Center, Dr. H. L. Trivedi Institute of Transplantation Sciences (IKDRC-ITS), Ahmedabad, India; 1 Department of Pathology, Laboratory Medicine, Transfusion Services and Immunohematology, Institute of Kidney Diseases and Research Center, Dr. H. L. Trivedi Institute of Transplantation Sciences (IKDRC-ITS), Ahmedabad, India

**Keywords:** Hemodialysis, hemodialysis catheter, persistent left superior vena cava

## Abstract

We report a case of end stage renal disease patient who displayed a persistent left superior vena cava (PLSVC) after placement of hemodialysis (HD) catheter through left internal jugular vein, as revealed by routine post-procedure X-ray chest. The diagnosis of PLSVC was confirmed by arterial blood gas, two-dimensional echocardiography, computed tomography thorax and angiographic examination. This anomaly is rather rare; few studies on safety of PLSVC for HD have been reported. The catheter was uneventfully used for HD for 2 months with careful continuous monitoring and removed after arteriovenous fistula was successfully cannulated. Physicians who place HD catheters in the left jugular/subclavian vein should be aware of the existence of PLSVC.

## Introduction

The most common clinical procedure performed by a nephrologist is placement of a non-cuffed dialysis catheter to obtain vascular access for immediate hemodialysis (HD) therapy.[1] HD vascular access catheters are essential for maintenance of HD, especially in situations where an immediate vascular access is required. These are increasingly used in patients with end stage renal disease (ESRD). Right internal jugular vein (IJV) is the preferred initial access site for catheter placement because of the relative direct path to superior vena cava (SVC) and right atrium, and the relative low incidence of central vein stenosis.[1] The second choice for placement of a catheter is not clear. Left internal jugular is a less desirable access site. The subclavian site allows excellent function but has a high rate of central vein stenosis and should therefore be avoided.[1] For single use or in patients confined to bed, femoral placement of a temporary dialysis catheter offers a convenient means for short-term vascular access. However, they are reported to be prone to higher infection rates and to loss of ambulation. Here, we report a case of successful insertion and use of HD catheter into the persistent left superior vena cava (PLSVC).

## Case Report

**Figure 1:**
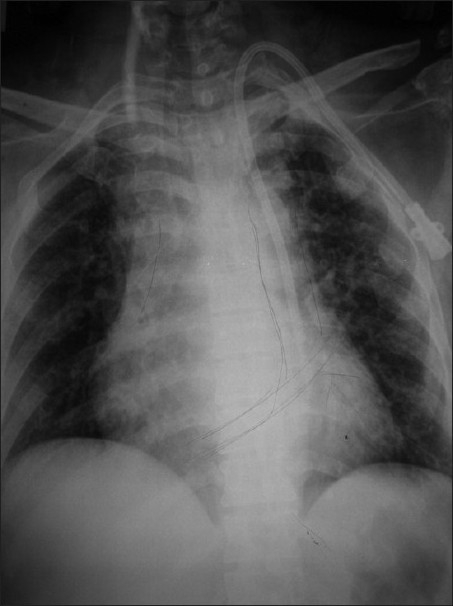
Chest radiograph showing the hemodialysis catheter passing through a persistent left superior vena cava

A 45-year-old female w ith ESRD due to hypertension and diabetes on maintenance HD for the last 6 months was admitted to our hospital for vascular access. She was diagnosed to have left radial arterio-venous (AV) fistula thrombosis. Thrombosed right IJV secondary to previous catheter placement was confirmed by Doppler ultrasonography. She lost her ambulation to femoral placement of a temporary dialysis catheter and was at high risk for femoral catheter site infection due to diabetic status. A dual-lumen cuffed dialysis catheter was inserted in the left IJV without complication except for mild resistance initially during guide wire insertion. Dark red blood return with non-pulsatile flow was noted immediately before insertion of the guide wire. Brisk dark (venous) blood return on aspiration of both catheter ports was noted. However, routine post-procedure X-ray chest [Figure 1] showed that dialysis catheter followed a left paramediastinal course from the left neck and repeated gentle aspiration of both ports resulted in brisk dark red blood return, which was sent for blood gas analysis. Blood gas samples drawn freely from the dialysis catheter and femoral vein were indistinguishable confirming venous blood (pH 7.29; PCO_2_ 62 mmHg; PO_2_ 39 mmHg; cHCO_3_ 28) and were different from femoral artery sample (pH 7.44; PCO_2_ 40 mmHg; PO_2_ 97 mmHg; cHCO_3_ 27 mmol/ L). A two-dimensional transthoracic echocardiogram demonstrated the dialysis catheter in the PLSVC, with the tip lying above the coronary sinus draining to the right atrium. An unusually large coronary sinus appeared as a dilated, echo-free space posteriorly in the atrio-ventricular (AV) groove between the left atrium and ventricle. Saline microbubble contrast was used to enhance the accuracy. The heart and great vessels were otherwise structurally unremarkable and the right SVC was present. Subsequent contrast angiography (venogram) with bolus contrast injection in the catheter confirmed that the catheter was placed in PLSVC draining in the right atrium. Computed tomography (CT) of the chest revealed the presence of a PLSVC with patent left innominate vein. The catheter was used successfully for HD treatments. HD sessions were performed with a blood flow rate of 250 ml/minute and without any difficulty. We decided to leave the catheter in place for HD due to nonavailability of other access sites. During the first HD, her vitals were maintained and the electrocardiogram (ECG) revealed no evidence of any arrhythmia/ischemia. Subsequently, the catheter was used uneventfully for HD for the next 2 months with careful and continuous monitoring. It was removed uneventfully once the AV fistula was successfully cannulated. There was no evidence of impaired venous drainage of the left upper limb. No complications attributable to the catheter were observed. She eventually underwent successful deceased donor renal transplantation.

## Discussion

PLSVC is the most common congenital anomaly of thoracic venous circulation, with incidences of 0.3% in healthy individuals and 4.5% in patients with congenital heart disease.[2,3] PLSVC is rarely reported because most dialysis catheters are inserted through the right IJV and PLSVC co-exists with the right SVC in >80% of patients.[4,5] PLSVC should be considered, especially when central venous catheterization via the left subclavian/IJV proves to be difficult and the fluoroscopy or chest X-ray suggests an aberrant left-sided course for the catheter. It is normally asymptomatic and hemodynamically insignificant.[6,8] PLSVC is detected incidentally during procedures such as HD catheter placement and can lead to serious complications like systemic embolization, provocation of arrhythmia, vascular thrombosis, shock, angina and cardiac arrest.[2,5,6,9,10] Echocardiography can accurately diagnose PLSVC non-invasively.[2] A lateral X-ray chest provides additional aid for correct diagnosis, in order to exclude cannulation of the superior intercostal vein or the accessory hemiazygos vein, which when cannulated, has a similar presentation on antero-posterior CXR with a cannulated PLSVC. PLSVC is recommended to be used safely for HD if the echocardiography confirms right atrial drainage, CT scan evidences a patent left innominate vein, ECG reveals no provocation of any arrhythmia and blood gas analysis confirms venous blood.[2,5] Dialysis catheter was uneventfully used for HD for 2 months with careful and continuous monitor as reported by others.[2,5,6,9] The presence of the catheter may trigger thrombosis of the coronary sinus, with possible detrimental consequences. Prospective trial should be carried out to determine the superiority of clopidogrel to prevent coronary sinus thrombosis, especially when catheter is required for a long time.

## Conclusion

PLSVC can be used safely for short-term HD, with careful and continuous monitoring. Physicians who place HD catheters in the left jugular or subclavian vein should be aware of the existence, diagnosis and complications of PLSVC to prevent misinterpretation of routine post-procedure X-ray chest, and unnecessary removal of dialysis catheters.
